# Advanced machine learning models for prediction of readmission and mortality risks in patients with chronic obstructive pulmonary disease using routine clinical data

**DOI:** 10.20407/fmj.2024-027

**Published:** 2025-04-17

**Authors:** Yasuhiro Goto, Daisuke Niwa, Shuhei Shibata, Ryoma Nishimoto, Masami Miyata, Takashi Kanno, Toshiyuki Washizawa, Masashi Kondo, Kazuyoshi Imaizumi

**Affiliations:** 1 Department of Respiratory Medicine, Fujita Health University, School of Medicine, Toyoake, Aichi, Japan; 2 Home Healthcare New Business Planning Department, Teijin Pharma Limited, Chiyoda-ku, Tokyo, Japan; 3 WASEDA University Ventures, Inc., Shinjuku-ku, Tokyo, Japan; 4 Global Business Services, IBM Japan, Ltd., Chuo-ku, Tokyo, Japan; 5 Development Office for Genomic Medical Research Center, Fujita Health University, School of Medicine, Toyoake, Aichi, Japan

**Keywords:** Chronic obstructive pulmonary disease, Machine learning, Risk prediction, Hospital readmission, Electronic health records

## Abstract

**Objectives:**

To develop a comprehensive machine learning model incorporating various clinical factors, including frailty and comorbidities, to predict 30-day readmission and mortality risk in patients with chronic obstructive pulmonary disease (COPD).

**Methods:**

This retrospective cohort study used electronic health records (EHR) from Fujita Health University Hospital (2004–2019) for 1294 patients with COPD and 3499 hospitalization or death events. The EHR contained longitudinal patient data (demographics, diagnoses, test results, clinical records). We developed two eXtreme Gradient Boosting models, the comprehensive Top64 and practical 11-feature models. We compared these with the Comorbidity, Obstruction, Dyspnea, and Previous Exacerbations index (CODEX) model, a widely used tool for predicting hospital readmission or death in patients with COPD. The area under the receiver operating characteristic curve (AUC) with 95% confidence interval (CI), sensitivity, and specificity were used to evaluate the model performance.

**Results:**

The Top64 (AUC: 0.769, 95% CI: 0.747–0.791) and practical 11-feature (AUC: 0.746, 95% CI: 0.730–0.762) models performed better than the CODEX model (AUC: 0.587, 95% CI: 0.563–0.611). The Top64 model showed 0.978 sensitivity and 0.341 specificity, and the practical 11-feature model achieved 0.955 sensitivity and 0.361 specificity. The calibration curves showed good agreement between the observed and predicted results for both models.

**Conclusions:**

A machine learning approach based on clinical data readily available from the EHR performed better than existing models in predicting 30-day readmission and mortality risks in patients with COPD. A comprehensive risk prediction tool may enhance individualized care strategies and improve patient outcomes in COPD management.

## Introduction

Chronic obstructive pulmonary disease (COPD), characterized by progressive airflow limitation and recurrent exacerbations, can lead to frequent hospitalizations and increased mortality rates.^1,2^ Hence, developing a COPD-specific risk prediction tool to identify patients at high risk for rehospitalization is crucial for implementing a personalized care plan.^3,4^ Various models for predicting readmission and death risks in patients with COPD have recently been proposed, with some using machine learning (ML) techniques.^5–7^ Compared with traditional statistical methods, the ML method has several advantages,^5,8^ including the ability to effectively handle complex nonlinear relationships between predictors and outcomes, as well as missing data and many explanatory variables. A predictive ML algorithm model using eXtreme Gradient Boosting (XGBoost) has demonstrated excellent performance in processing missing data without imputation and is particularly useful for developing predictive models based on diverse clinical data.^9–12^ Furthermore, the Shapley Additive exPlanations (SHAP) approach enhances the interpretability of the ML model by revealing the influence and direction of individual factors on clinical outcomes.^13–15^

Because the prevalence of COPD is higher among older adults and COPD is often accompanied by many other diseases and frailty in this population, developing a more comprehensive approach to risk prediction is necessary.^2,16,17^ However, the indicators developed to predict the risk of COPD exacerbation are mainly based on demographic factors, such as the analysis of insurance claim databases and factors specific to the respiratory system, such as respiratory function tests.^18–21^ A previous study developing a prediction model for 30-day rehospitalization with machine learning using patients’ claims data^6^ showed only slight improvement in performance (area under the receiver operating characteristic curve [AUC] of 0.65) compared with models based on conventional indices, such as the LACE (length of stay, acuity of admission, comorbidities, emergency room visits).^22^ Incorporating various clinical factors such as frailty^7,23^ and comorbidities^5^ that have been suggested to be involved may improve the risk prediction model. In the present study, we aimed to develop a comprehensive predictive model for rehospitalization and mortality risks in patients with COPD 30 days after discharge by incorporating missing data and large amounts of clinical data obtained in daily clinical practice, such as factors related to frailty and complications, which were previously difficult to handle using conventional statistical methods and advanced ML technologies. Additionally, the impact of features on outcomes and individual patient risk factors was described visually.

## Methods

### Study design and data source

This retrospective cohort study used electronic health records (EHR) from the Japanese Fujita Health University Hospital between May 17, 2004, and December 10, 2019. This study was approved by the Certified Review Board of Fujita Health University (approval number: HM20-482, approval date: March 26, 2021) and was conducted in compliance with the Declaration of Helsinki. Patient consent was not required because this was a retrospective observational study in which anonymized data were used.

### Study population

**Figure 1:**
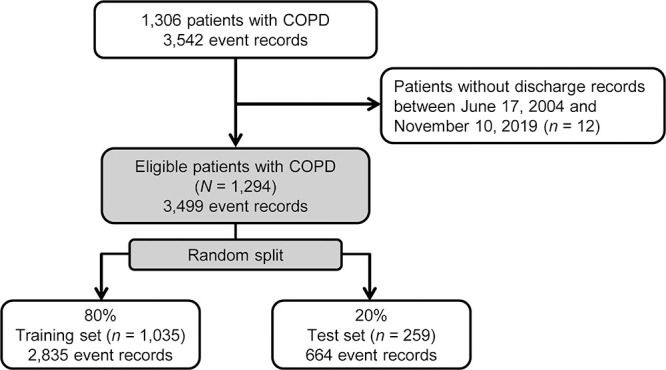
Flow diagram showing the disposition of patients with COPD. Number of event records represents total number of hospitalization or death records. Abbreviations: COPD, chronic obstructive pulmonary disease.

The inclusion criteria were as follows: 1) International Classification of Diseases, Tenth Revision code for COPD within 30 days before discharge (J42-44); 2) discharge between June 17, 2004, and November 10, 2019; and 3) available 30-day post-discharge follow-up data. The exclusion criterion was a lack of records for blood tests or vital signs within 30 days of discharge from the hospital. Because this was a retrospective study, we analyzed approximately 1000 cases collected during the study period. We determined that this sample size was adequate for our analyses; the number of cases in previous studies ranged from 900 to 2700.^20–22,24^ We included 1294 eligible patients, accounting for 3499 hospitalizations or death events (Figure 1). The dataset was randomly divided into 80% training and 20% test sets on a patient-by-patient basis to ensure that hospital records for the same patient were not divided between sets (Supplementary Figure 1). The cohort was divided into training and test sets of 1035 patients (2835 events) and 259 patients (664 events), respectively.

### Outcome and predictor variables

**Figure 2:**
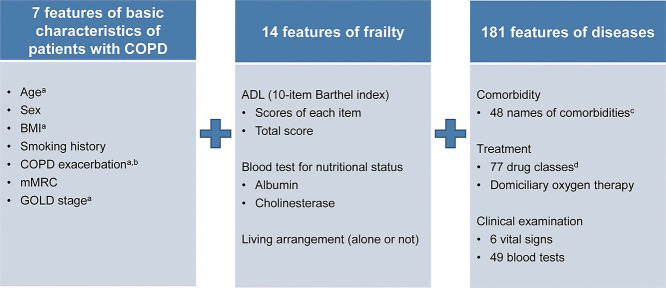
Candidate predictors (202 features) belonged to three categories. The latest data before discharge from the hospital were used for all variables. Data on vital signs or blood tests were limited to measurements obtained within 30 days of discharge from the hospital. If one patient had multiple data points on the same day, the average of the data was used. Although many data, such as blood test results and vital signs, were continuous variables, the stage and code were discrete variables. Abbreviations: ADL, activities of daily living; BMI, body mass index; COPD, chronic obstructive pulmonary disease; GOLD, Global Initiative for Chronic Obstructive Lung Disease; mMRC, modified Medical Research Council; CODEX, Comorbidity, Obstruction, Dyspnea, and Previous Exacerbations index. ^a^ Age, BMI,^20^ COPD exacerbation, and GOLD stage^2^ were determined from collected data. ^b^ COPD exacerbation was defined as “the number of hospitalizations for COPD exacerbation,” with reference to the definition of exacerbation, severe exacerbations of COPD during the previous year (hospitalizations or emergency department consultations), in the CODEX index. ^c^ Comorbidities were classified into 48 categories with reference to International Classification of Diseases, Tenth Revision codes. ^d^ Prescribed drugs were classified into 77 groups using YJ codes.

We developed a comprehensive machine learning model that incorporates various clinical factors, including frailty and comorbidities at the time of admission, to predict the risk of readmission or death within 30 days of discharge in patients with COPD. The primary outcome was hospital readmission or death within 30 days of discharge and was compared with no readmission or survival during the same period. For patients who were hospitalized more than once, the data were divided for each hospitalization, and each discharge day was used as the index date (Supplementary Figure 1). We examined the risk of readmission or death within 30 days of hospitalization for COPD. Therefore, the outcome group included both readmission and death; the control group comprised patients with no readmission and who survived during the same period. We categorized death as readmission owing to the inability to track all specific causes of death and the potential overlap between severe deterioration leading to death and readmission. A wide range of potential predictive variables, including tests commonly performed in routine clinical practice, were extracted from the EHR database. Variables for which data from >90% of patients were missing (COPD assessment test) were excluded. Finally, 202 potential predictive variables were extracted (Figure 2) and classified as follows: 1) basic features of COPD (seven features), 2) frailty (14 features), and 3) disease-related factors (181 features).

### Model development and statistical analysis

**Figure 3:**
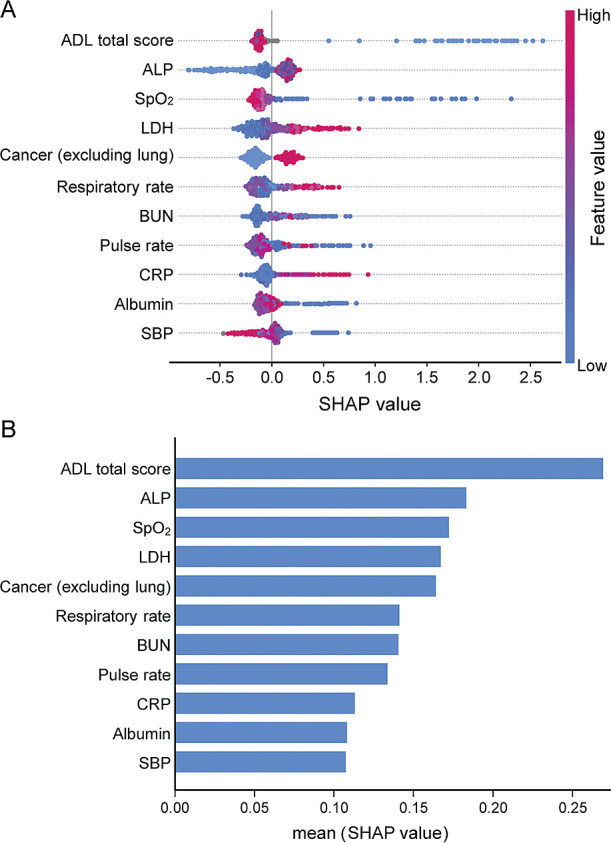
Summary of SHAP analysis of the practical model showing the 11 most important features and their impact on model output. Features included in the “practical model” were ranked in descending order based on SHAP value; features at the top of the graph contribute more to the prediction model. Impact on the model output (A). The x-axis represents the SHAP value with 0 as the reference, where the contribution to the outcome is positively larger to the right (increasing risk) and negatively larger to the left (decreasing risk). The y-axis (color of the point) represents the feature value, where red is larger and blue is smaller. For example, SpO_2_ has several blue dots to the right of 0, indicating that a low SpO_2_ level indicates an increased risk. By contrast, CRP has several red dots to the right of 0, meaning a high CRP level is associated an increased risk. Average impact on model output magnitude (B). Abbreviations: CRP, C-reactive protein; SHAP, SHapley Additive exPlanations; SpO_2_, oxygen saturation. The abbreviations and notes for these features are listed in Table 1. ^a^ Plots are relative to all data, not “low” or “high” compared with the reference value.

**Figure 4:**
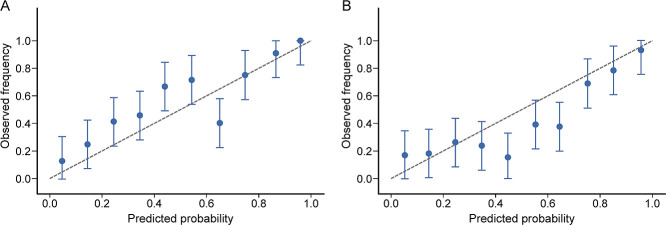
Calibration curves for the Top64 model (A) and practical model (B) for predicting 30-day hospital readmission or mortality in patients with COPD in the test and training sets. Error bars represent 95% confidence intervals. Abbreviations: COPD, chronic obstructive pulmonary disease.

In this study, we used XGBoost to develop our predictive models using a training dataset comprising 1035 patients. Five-fold cross-validation was performed exclusively on the training data to optimize the model parameters and select relevant features, thereby preventing information leakage. From the training process, we calculated the AUC, sensitivity, and specificity to evaluate the model performance. Variable importance and contributions were assessed using SHAP values to interpret the influence of each predictor on the outcomes. Subsequently, we evaluated the final model’s performance on an independent test dataset containing 259 patients, which was not involved in the training or cross-validation processes. Performance metrics, including the AUC and precision-recall AUC, were calculated for the test set to assess the model’s generalizability (see Supplementary Figures 2, 3). Figure 3 and Supplementary Figures 2 and 3 present the results derived from the training data set, while Figure 4 illustrates the combined performance evaluation using both training and test data sets.

The first XGBoost algorithm (ver. 1.3.3)^9^ was used to develop a risk prediction model to effectively handle missing data. All 202 features, categorized into basic patient characteristics, frailty indicators, and comorbidities, were initially input into the XGBoost model. To reduce potential noise from the large number of features, we applied a feature selection procedure aimed at minimizing the feature set while preserving model accuracy. Specifically, we used the default feature importance ranking provided by XGBoost to select the top 64 features. This reduction was carried out iteratively, with model performance metrics such as AUC and precision-recall AUC used to determine the optimal number of features. Ultimately, the feature set was reduced from 202 to 64 based on AUC. The selected features were then used to develop the final model, and their performance was evaluated using AUC and precision-recall AUC (Supplementary Figure 2). The performance of the model was assessed using the AUC, sensitivity, specificity, and 95% confidence interval (CI). Five-fold cross-validation was performed for internal validation. The final AUC was calculated as the mean value obtained from the test set. A reference model based on the Comorbidity, Obstruction, Dyspnea, and Previous Exacerbations index (CODEX model),^21^ which is commonly used to predict hospital readmission and death in patients with COPD, was compared with the constructed model in terms of performance. On the basis of this comparison, we evaluated the improvement in performance using the ML approach compared with that of traditional tools. Logistic regression analysis was performed for each variable set in our model and the CODEX model to assess the performance differences between traditional statistical methods and ML approaches. A test set was used to assess the model calibration. SHAP (ver. 37.0)^13^ was used to illustrate and describe the effects of feature quantities on each patient’s model outputs and risk factors.

### Model optimization and feature selection

We developed two main models: 1) a comprehensive model including the 64 leading features (Top64 model) and 2) a practical model with 11 features having the highest ranks (practical 11-feature model). Feature selection was repeated while minimizing its effect on the accuracy of the model.

All analyses were performed using Python^TM^ (ver. 3.8.5, Python Software Foundation, Beaverton, OR, USA).

## Results

### Patient characteristics

Table 1 presents the baseline characteristics of the included patients. Patients’ mean age and body mass index (BMI) were 74.4 years and 20.7 kg/m^2^, respectively. Notably, most patients were male (80.4%) and had a history of smoking (83.8%).

### Model development and feature selection

Using XGBoost, we reduced the number of features and calculated the AUC and precision recall-AUC. Consequently, an optimized model with selected 64 features (the Top64 model) was chosen.

SHAP analysis was performed to rank the 64 features of this model according to their contributions (Supplementary Figure 3, Supplementary Table 1). There are too many features in the 64 feature set, making it difficult to use in actual clinical practice. Therefore, we added the top features and used the Top 64 model with the smallest number of features (n=11) that most closely approximated AUC, sensitivity, and specificity, as the practical model. The top 11 features were selected to construct a “practical model” for usability in a clinical setting (Figure 3, Table 2). These key features included activities of daily living (ADL), alkaline phosphatase, oxygen saturation, lactate dehydrogenase, non-lung cancer, respiratory rate, blood urea nitrogen, pulse rate, C-reactive protein, albumin, and systolic blood pressure.

### Model performance

Additional variables in each category were added sequentially to improve model performance (Table 3). The Top64 model exhibited an AUC, sensitivity, and specificity of 0.769 (95% CI, 0.747–0.791), 0.978 (0.927–0.984), and 0.341 (0.304–0.377), respectively. The practical model with 11 features had similar results, with AUC, sensitivity, and specificity of 0.746 (95% CI, 0.730–0.762), 0.955 (0.945–0.965), and 0.361 (0.328–0.394), respectively. The CODEX model exhibited an AUC, sensitivity, and specificity of 0.587 (95% CI, 0.563–0.611), 0.999 (0.999–1.000), and 0.007 (0.000–0.014), respectively.

Calibration curves for the Top64 and practical models showed good agreement between observed and predicted outcomes (Figure 4).

### Individual patient risk factors

**Figure 5:**
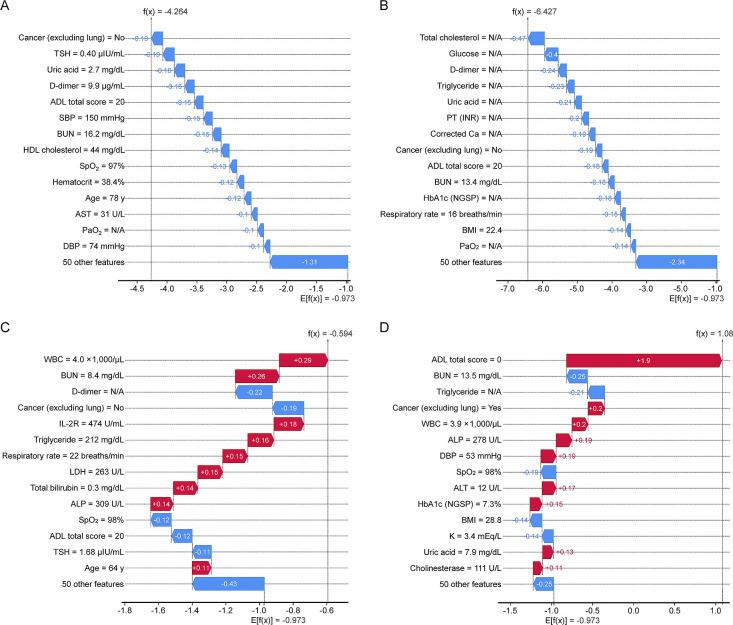
Illustration of predictions of typical instances using the SHAP values. For each patient with COPD, the 14 features with the greatest contribution and 50 other features are presented in order from the top. SHAP values for each feature are shown for two patients at low risk, (A) and (B), and two at high risk, (C) and (D). The red bar indicates the direction of increasing risk and the blue bar indicates the direction of decreasing risk. For instance, the comorbidity “cancer (excluding lung)” was a protective factor (blue color) for patients A to C because they did not have cancer (=No) but a risk factor (red color) for patient D who had cancer (=Yes). Total ADL score contributes to the decreasing risk for patients A to C (total score=20) but contributes to the increasing risk for patient D (=0). The abbreviations and notes for these features are provided in Supplementary Table 1. Not available (N/A) indicates missing data. Abbreviations: ADL, activities of daily living; COPD, chronic obstructive pulmonary disease; SHAP, SHapley Additive exPlanations.

A typical example of a patient was used in the SHAP analysis, and the contribution of each individual characteristic was interpreted using a single model (Figure 5). In two patients (A and B) with low risk, all features were blue, i.e., in the direction of decreasing risk. However, many features were red, i.e., in the direction of increasing risk, in two patients (C and D) with high risk. Because the modeling of SHAP values considers the interactions between variables, the degree and direction (increasing/decreasing risk) of the feature’s impact vary among patients. The same features showed different risks based on the condition of each patient.

## Discussion

In the present study, we developed a high-performance model for predicting 30-day rehospitalization and death risks in patients with COPD using only data that are readily available in routine clinical practice. This approach is distinct from previous methods, which often relied on specialized testing and complex assessments.^5–7^ An ML approach was used to integrate a wide range of routine clinical data, including traditional respiratory measures, comorbidities, frailty markers, standard blood tests, and basic vital signs, to build the Top64 and practical 11-feature models.

Adding the features of frailty (AUC, 0.662) and disease (AUC, 0.765) to the basic features of patients with COPD (AUC, 0.533) significantly improved performance of the model. All seven cardinal features of patients with COPD (age, sex, BMI, smoking history, exacerbations, modified Medical Research Council [dyspnea scale], and Global Initiative for Chronic Obstructive Lung Disease [GOLD] stage) were commonly used prognostic variables in patients with COPD^3,21^. However, none of the 11 most important features in the present study were included. COPD aggravation and GOLD stage were included in the Top64 model, and their SHAP rankings were not high (32 and 62, respectively). Notably, the 11 most important predictors were traits in the frailty and comorbidity categories. Low ADL levels, a hallmark of frailty, contributed the most to the outcome in the present study. Furthermore, low serum albumin levels, another characteristic of frailty, indicates malnutrition. In a previous study, undernutrition was reported to be a risk factor for poor COPD prognosis.^7,9^ In a study conducted to identify risk factors for COPD,^8^ frailty measured using patient-reported outcomes was the best predictor of 30-day readmission, based on univariate logistic regression analysis.

The Top64 and practical models in our study exhibited AUC values of 0.769 (95% CI, 0.747–0.791) and 0.746 (0.730–0.762), respectively. These values exceeded those of the CODEX model (0.587; 95% CI, 0.563–0.611). In an original CODEX study by Almagro et al., an AUC of 0.73 (95% CI, 0.70–0.76) was reported for predicting 90-day mortality or rehospitalization.^21^ A direct comparison is difficult owing to differences in study populations and evaluation timelines. Nevertheless, despite the shorter 30-day prediction window, both the Top64 and practical 11-feature models performed better than the CODEX model and exceeded the performance reported in the original CODEX study. This finding underscores the importance of our ML approach and use of a comprehensive set of readily available clinical data, which resulted in robust predictive performance of our model over both previously reported models and the CODEX model.

The Top64 and practical models used in the present study had high sensitivity (0.978 and 0.955, respectively) and low specificity (0.341 and 0.361, respectively). A risk prediction model for 30-day readmission has been designed to identify high-risk candidates, for individualized interventions and improve prognosis.^5–7,25,26^ The key objective of our model was to identify as many high-risk patients as possible at discharge rather than to identify patients who were more likely to not be readmitted. Furthermore, a practical model that reduces the number of variables from the Top64 model to 11 may be a useful tool in clinical settings.^7^

Our model also outperformed recent ML approaches. In a study by Goto et al., in which patient-billed Diagnosis Procedure Combination data were used, an AUC of 0.662 (95% CI, 0.646–0.677) was reported for predicting 30-day readmission. Regarding performance, an AUC of 0.605 (95% CI, 0.589–0.620) was observed when compared with previous indicator models, such as the LACE index.^5^ Notably, in the present study, we used more detailed clinical data, including comprehensive blood test results and vital signs. This rich dataset provides a more comprehensive presentation of each patient’s condition and may have contributed to the excellent performance of our model.

COPD should be understood as a complex systemic disease in which not only respiratory function but also multiple factors influence patient outcomes.^20,27^ However, previous studies have relied on respiratory-specific factors and limited demographic data to predict the risk associated with COPD.^20,22,28^ Therefore, these approaches have often failed to capture the complex and multifaceted nature of COPD, particularly its association with polymorbidity and frailty in older adults.^29,30^ Notably, a comprehensive risk prediction model incorporating various clinical data including those associated with frailty and comorbidities, which has not been fully exploited in previous studies, was developed in the present study using ML technologies and incorporating a wide range of blood test results and vital signs.

A key innovation in this comprehensive approach is the ability to extract meaningful patterns from routine clinical data. These data are readily available for standard patient care; however, previous predictive models have been underused. Advanced ML algorithms, particularly XGBoost, have effectively addressed the complexity and intrinsic deficits of real clinical data.^9,31^ Therefore, a more detailed and accurate prediction tool that reflects the systemic features of COPD has been developed. This approach enhances the predictive performance of the model and is consistent with the growing recognition that COPD is a complex and systemic disease requiring a holistic management approach.^32,33^

A strength of this study lies in the use of individual diseases and test results as predictors to assess the contribution of each variable to outcomes and identify individual risk factors using SHAP. Comorbidities are typically summarized using the Charlson Comorbidity Index^34^ in CODEX^21^ and other tools^3,13,18^, making it challenging to interpret the specific risk associated with each disease. By examining the contribution of patient-specific risk factors to outcomes, our findings provide valuable reference information for personalized care. The developed models are expected to support decision-making for individualized intervention strategies after hospital discharge in clinical settings. Another notable strength is that the prediction models, based on EHR, only used predictors commonly available in routine practice, thereby enabling the timely assessment of a patient’s risk at discharge.^35^ The success of our model highlights an unexploited potential for routine clinical information. ML technologies may be highly effective in revealing the complex relationships between diverse clinical factors and patient outcomes, which may not be revealed using traditional statistical methods.

This study also has some limitations. First, this was a retrospective study conducted at a single center, which may limit generalizability of our findings. Our model showed promising results; however, these may vary depending on the health care setting and patient attributes and should be validated in diverse patient populations and patient care settings across multiple centers.^19,22,23^ Second, the EHR may include data gaps, variations in measurement frequency and intervals, and patients who were lost to follow-up. The COPD assessment test is a potential predictor^36^; we excluded this because <10% of our patients had such data. Further examination is needed, including external verification of patients with COPD from other regions and other medical institutions.^24,37^

In conclusion, our study revealed the feasibility and potential of using ML approaches with routine clinical data to develop accurate predictive models for the risk of readmission and mortality in patients with COPD. We developed a model that outperforms existing risk prediction tools by incorporating a comprehensive range of clinical factors and leveraging advanced ML techniques. Our findings will facilitate more personalized and proactive management strategies for COPD care, potentially reducing readmission rates and improving patient outcomes. Future multicenter prospective studies are required to validate and refine these models and ultimately translate our findings into improved clinical practice and patient care.

## Figures and Tables

**Table 1:** Demographic and clinical characteristics of patients with COPD

Characteristic	Patients with COPD (N=1294)	
		n
Age, years	74.4±9.8	1294
Sex, male	1041 (80.4)	1294
BMI, kg/m^2^	20.7±4.5	1240
FEV_1_%	57.6±15.1	924
GOLD stage	1.6±0.8	910
mMRC	1.4±1.1	181
Charlson Comorbidity Index	6.1±3.8	1294
Hospitalization^a^	2.7±2.7	1294
COPD exacerbation^b^	1.6±2.4	1294
Smoking history	853 (83.8)	1018
ADL total score (10-item Barthel Index)	14.5±8.0	1116
ALP, U/L	318.1±297.3	1137
SpO_2_, %	93.9±10.8	1165
LDH, U/L	276.4±292.6	1125
Cancer (excluding lung cancer)	513 (40.0)	1294
Lung cancer	306 (23.6)	1294
Respiratory rate, breaths/min	19.5±10.4	1014
BUN, mg/dL	23.8±22.6	1225
Pulse rate, beats/min	75.4±24.3	1288
CRP, mg/dL	3.58±5.51	1187
Albumin, g/dL	3.17±0.71	1186
Systolic blood pressure, mmHg	112.8±24.9	1288

Abbreviations: ADL, activities of daily living; ALP, alkaline phosphatase; BMI, body mass index; BUN, blood urea nitrogen; COPD, chronic obstructive pulmonary disease; CRP, C-reactive protein; FEV_1_%, forced expiratory volume % in 1 second; GOLD, Global Initiative for Chronic Obstructive Lung Disease; LDH, lactate dehydrogenase; mMRC, modified Medical Research Council; SpO_2_, oxygen saturation. Data are presented as mean±standard deviation or n (%). ^a^ Number of hospitalizations for any cause. ^b^ Number of hospitalizations for COPD exacerbations.

**Table 2:** Direction of the contribution of each feature to the outcome

SHAP rank	Feature	Category	Note	Direction associated with increased risk^a^
1	ADL total score	Frailty	Total score of the 10-item Barthel index	Low
2	ALP	Diseases: Clinical examination	Blood biomarker related to liver and renal functions	High
3	SpO_2_	Diseases: Clinical examination	Vital sign	Low
4	LDH	Diseases: Clinical examination	Blood biomarker related to liver and renal functions	High
5	Cancer (excluding lung)	Diseases: Comorbidity	Name of comorbidity	Yes
6	Respiratory rate	Diseases: Clinical examination	Vital sign	High
7	BUN	Diseases: Clinical examination	Blood biomarker related to liver and renal functions	—
8	Pulse rate	Diseases: Clinical examination	Vital sign	—
9	CRP	Diseases: Clinical examination	Blood biomarker related to Inflammation	High
10	Albumin	Frailty	Blood test for nutritional status	Low
11	SBP	Diseases: Clinical examination	Vital sign	Low

Abbreviations: SHAP, Shapley Additive exPlanations; ADL, activities of daily living; ALP, alkaline phosphatase; BUN, blood urea nitrogen; CRP, C-reactive protein; LDH, lactate dehydrogenase; SpO_2_, oxygen saturation; SBP, systolic blood pressure.

**Table 3:** Performance of different prediction models for 30-day hospital readmission or mortality after discharge

Patients with COPD (N=1294) Model	No. of features	AUC (95% CI)	Sensitivity (95% CI)	Specificity (95% CI)
Basic characteristics of patients with COPD	7	0.533 (0.512–0.554)	0.925 (0.912–0.939)	0.115 (0.074–0.156)
Basic characteristics of patients with COPD +Frailty	21	0.662 (0.647–0.676)	0.951 (0.935–0.966)	0.294 (0.274–0.314)
Basic characteristics of patients with COPD +Frailty +Diseases (comorbidity)	69	0.694 (0.682–0.707)	0.961 (0.950–0.972)	0.304 (0.280–0.328)
Basic characteristics of patients with COPD +Frailty +Diseases (comorbidity and treatment)	147	0.707 (0.690–0.724)	0.965 (0.955–0.975)	0.303 (0.268–0.339)
Basic characteristics of patients with COPD +Frailty +Diseases (comorbidity, treatment, and clinical examination)	202	0.765 (0.745–0.786)	0.979 (0.97–0.984)	0.338 (0.301–0.374)
Top64 model	64	0.769 (0.747–0.791)	0.978 (0.927–0.984)	0.341 (0.304–0.377)
Practical model	11	0.746 (0.730–0.762)	0.955 (0.945–0.965)	0.361 (0.328–0.394)
CODEX model^a^	4	0.587 (0.563–0.611)	0.999 (0.999–1.000)	0.007 (0.000–0.014)

Abbreviations: CI, confidence interval; COPD, chronic obstructive pulmonary disease; FEV_1_%, forced expiratory volume % in 1 second; mMRC, modified Medical Research Council; AUC, area under the receiver operating characteristic curve. The 95% CIs were calculated using 1.96×standard error. The AUC, sensitivity, and specificity were calculated using the training data set and k-fold cross-validation. ^a^ In the CODEX model, the age-adjusted Charlson Comorbidity Index, FEV_1_%, mMRC, and COPD exacerbation were used as explanatory variables with reference to items of the CODEX index. The age-adjusted Charlson Comorbidity Index was calculated from the collected data.

## Data Availability

Data supporting the findings of this study are available from the corresponding author upon reasonable request.
