# Supplementary material for: Advanced machine learning models for prediction of readmission and mortality risks in patients with chronic obstructive pulmonary disease using routine clinical data
Source: Fujita Med J. 2025 Apr 17;11(3):121–8. doi: 10.20407/fmj.2024-027 (PMC12327213; doi:10.20407/fmj.2024-027)
Supplement: Supplementary file 1 — Supplementary Materials [file fmj-11-121-s001.pdf]

Supplementary Materials

Supplementary Figure 1. Example of how to divide and count the patient's records for each hospitalization.

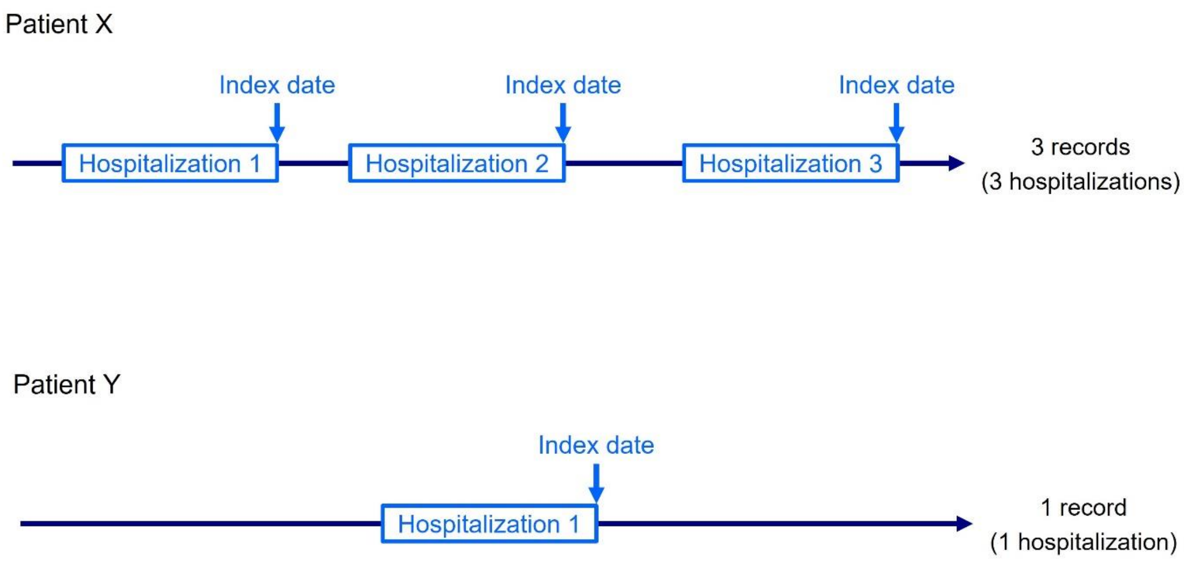

Patient X was hospitalized thrice, and an index date was assigned to the discharge date of each hospitalization; therefore, the data were divided into three records. Patient Y was hospitalized once; the data were counted as one record. When splitting the data, the records were divided according to patient to prevent splitting of multiple hospitalization records of the same patient.

Supplementary Figure 2. Process of feature selection using XGBoost.

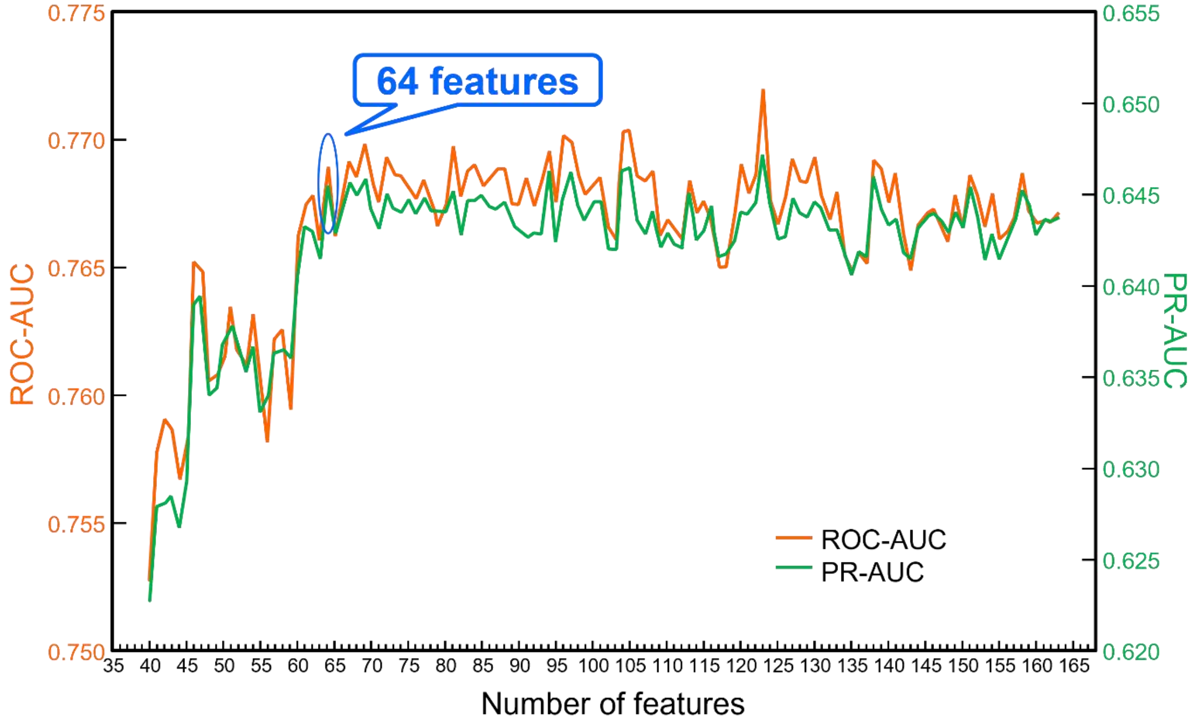

Each model with a number of features ranging from 163 to 40 was built using XGBoost. We chose a model in which the ROC-AUC and PR-AUC did not decrease and the number of features was the smallest. An optimal model with selected 64 features (the "Top64 model") was obtained.

Abbreviations: PR-AUC, precision recall-area under the curve; ROC-AUC, receiver operating characteristic-area under the curve; XGBoost, eXtreme gradient boosting.

Supplementary Figure 3. Summary of SHAP analysis of the Top64 model showing the 64 features and their impact on the model output.

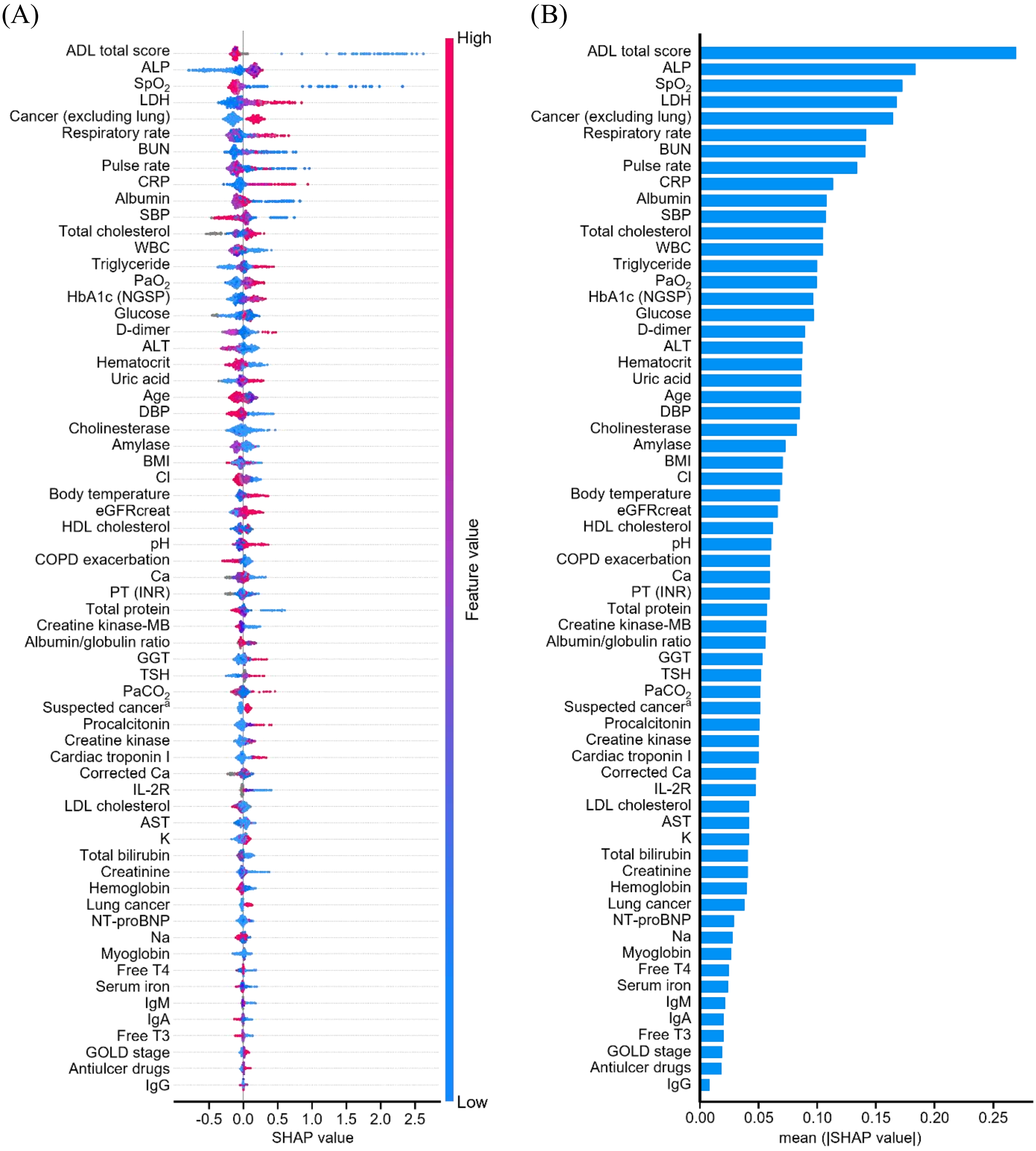

The features included in the "Top64 model" were ranked in descending order based on the SHAP value; the features at the top of the graph contribute more to the prediction model. Impact on the model output (**A**); the x-axis represents the SHAP value with 0 as the reference, where the contribution to the outcome is positively larger to the right (increasing risk) and negatively larger to the left (decreasing risk). The y-axis (color of the point) represents the feature value, where red is larger, and blue is smaller. Average impact on the model output magnitude (**B**).

Abbreviations: SHAP, SHapley Additive exPlanations. The abbreviations and notes for the features are provided in Supplementary Table 1.

[a] "Suspected cancer" refers to suspected cancer, excluding lung cancer.

Supplementary Table 1. Selected 64 features for the optimized "Top 64 model" with SHAP ranking

Category	Feature	SHAP rank	Note
Basic characteristics of patients with COPD	Age	22	
	BMI	26	
	COPD exacerbation	32	Number of hospitalizations for COPD exacerbation
	GOLD stage	62	GOLD classification of COPD severity
Frailty	**ADL total score**[a]	**1**	Total score of the 10-item Barthel index
	**Albumin**[a]	**10**	Blood test for nutritional status
	Cholinesterase	24	
Diseases: Comorbidity	**Cancer (excluding lung)**[a]	**5**	Name of comorbidity
	Suspected cancer (excluding lung)	41	
	Lung cancer	53	
Diseases: Treatment	Antiulcer drugs	63	Drug class
Diseases: Clinical examination	**SpO_2**[a]	**3**	Vital sign
	**Respiratory rate**[a]	**6**	
	**Pulse rate**[a]	**8**	
	**SBP**[a]	**11**	
	DBP	23	
	Body temperature	28	
	**ALP**[a]	**2**	Blood biomarker related to liver and renal functions
	**LDH**[a]	**4**	
	**BUN**[a]	**7**	
	ALT	19	
	eGFRcreat	29	
	Total protein	35	
	Albumin/globulin ratio	37	
	GGT	38	
	AST	48	
	Total bilirubin	50	
	Creatinine	51	
	**CRP**[a]	**9**	Blood biomarker related to inflammation
	WBC	13	
	IgM	59	
	IgA	60	
	IgG	64	
	Total cholesterol	12	Blood biomarker related to lifestyle-related disease
	Triglyceride	14	
	HbA1c (NGSP)	16	
	Glucose	17	
	Uric acid	21	
	HDL cholesterol	30	
	LDL cholesterol	47	
	PaO_2	15	Blood gas

Category	Feature	SHAP rank	Note
	pH	31	
	$PaCO_2$	40	
	D-dimer	18	Blood biomarker related to cardiovascular diseases
	PT (INR)	34	
	Creatine kinase-MB	36	
	Creatine kinase	43	
	Cardiac troponin I	44	
	NT-proBNP	54	
	Myoglobin	56	
	Hematocrit	20	Blood biomarker related to anemia
	Hemoglobin	52	
	Serum iron	58	
	Cl	27	Blood electrolytes
	Ca	33	
	Corrected Ca	45	
	K	49	
	Na	55	
	TSH	39	Blood biomarker related to thyroid functions
	Free T4	57	
	Free T3	61	
	Amylase	25	Others
	Procalcitonin	42	
	IL-2R	46	

Abbreviations: ADL, activities of daily living; ALP, alkaline phosphatase; ALT, alanine aminotransferase; AST, aspartate aminotransferase; BMI, body mass index; BUN, blood urea nitrogen; COPD, chronic obstructive pulmonary disease; CRP, C-reactive protein; DBP, diastolic blood pressure; eGFRcreat, estimated glomerular filtration rate calculated based on creatinine; free T3, free triiodothyronine; free T4, free thyroxine; GGT, γ-glutamyl transpeptidase; GOLD, Global Initiative for Chronic Obstructive Lung Disease; HbA1c (NGSP), glycated hemoglobin (National Glycohemoglobin Standardization Program); HDL, high-density lipoprotein; IL-2R, interleukin-2 receptor; LDH, lactate dehydrogenase; LDL, low-density lipoprotein; NT-proBNP, N-terminal prohormone of brain natriuretic peptide; $PaCO_2$, partial pressure of arterial carbon dioxide; PaO_2, partial pressure of arterial oxygen; PT (INR), prothrombin time (international normalized ratio); SBP, systolic blood pressure; SHAP, SHapley Additive exPlanations; SpO_2, oxygen saturation; TSH, thyroid-stimulating hormone; WBC, white blood cell count.

[a] The boldfaced features represent the 11 highest-ranked features selected for the "practical model."
